# Sodium thiosulfate does not affect energy metabolism or organ (dys)function during resuscitation from murine trauma-and-hemorrhage

**DOI:** 10.1186/s40635-025-00778-0

**Published:** 2025-08-06

**Authors:** Maximilian Feth, Mirabel Gracco, Michael Gröger, Melanie Hogg, Sandra Kress, Andrea Hoffmann, Enrico Calzia, Ulrich Wachter, Peter Radermacher, Tamara Merz

**Affiliations:** 1 Department of Anesthesiology, Critical Care, Emergency Medicine and Pain Therapy, German Armed Forces Hospital Ulm, Oberer Eselsberg 40, 89081 Ulm, Germany; 2 Institute for Anesthesiological Pathohysiology and Process Engineering, Ulm University Hospital, Helmholtzstrasse 8/1, 89081 Ulm, Germany; 3 Département de médecine intensive – reanimation et médecine hyperbare, Centre Hospitalier Universitaire, 49933 Angers Cedex 9, France; 4 Department of Anesthesiology and Intensive Care Medicine, University Hospital, 89081 Ulm, Germany

**Keywords:** H_2_S, Oxidative stress, Nitrosative stress, Mitochondrial function, Stable isotopes, Metabolism

## Abstract

**Background:**

In murine models, controversial data have been reported on the effect of hydrogen sulfide (H_2_S) administration during resuscitation from trauma-and-hemorrhage. The H_2_S donor sodium thiosulfate (Na_2_S_2_O_3_) is a recognized drug devoid of major side effects, and, hence, we determined its effects in our full scale ICU-model of resuscitated murine trauma-and-hemorrhage. We hypothesized that Na_2_S_2_O_3_ might improve energy metabolism and thereby exert organ-protective effects as previously demonstrated in animals with genetic cystathionine-γ-lyase (CSE) deletion (CSE^−/−^).

**Methods:**

30 mice underwent combined blast wave-induced blunt chest trauma followed by 1 h of hemorrhagic shock (mean arterial pressure MAP = 35 ± 5 mmHg). Thereafter, resuscitation was initiated comprising re-transfusion of shed blood, lung-protective mechanical ventilation, fluid resuscitation and continuous i.v. noradrenaline infusion to maintain MAP > 55 mmHg over 6 h, and randomized administration of either i.v. 0.45 mg/g_bodyweight_ Na_2_S_2_O_3_ or vehicle (NaCl 0.9%). Hemodynamics, lung mechanics, gas exchange, acid–base-status and organ function parameters were recorded. Metabolic pathways were quantified based on gas chromatography/mass spectrometry assessment of plasma isotope enrichment during primed-continuous infusion of stable, non-radioactive, isotope labeled substrates. Mitochondrial function was determined using high-resolution respirometry, and tissue target proteins (nitrotyrosine formation, extravascular albumin accumulation, CSE expression) were analyzed using immunohistochemistry.

**Results:**

Data originate from 23 mice (Na_2_S_2_O_3_ *n* = 12; vehicle *n* = 11)_._ Na_2_S_2_O_3_ affected neither survival nor noradrenaline requirements. While minute ventilation had to be increased over time in both groups to maintain arterial PCO_2_ without intergroup difference, arterial PO_2_ decreased over time in Na_2_S_2_O_3_-treated mice (*p* = 0.006). Although arterial pH decreased in both groups (vehicle *p* = 0.049; Na_2_S_2_O_3_ *p* < 0.001), metabolic acidosis was more pronounced in the Na_2_S_2_O_3_ group. Neither metabolic pathways nor tissue mitochondrial respiratory activity or tissue target proteins showed any intergroup differences.

**Discussion:**

In this model of resuscitated trauma-and-hemorrhage, Na_2_S_2_O_3_ did not exert any beneficial metabolic or organ-protective effect and was even associated with impaired pulmonary function. These results are in contrast to our previous findings in CSE^−/−^ mice, but in line with more recent findings in CSE^−/−^ mice with pre-existing comorbidities. Hence, our studies do not support a beneficial role of Na_2_S_2_O_3_ in trauma resuscitation.

## Introduction

After trauma-and-hemorrhage, restoring tissue perfusion represents an ischemia/reperfusion (I/R) injury leading to enhanced formation of reactive oxygen and nitrogen species [1, 2], ultimately causing multiple organ failure, among others, as a result of mitochondrial dysfunction [3–5]. This effect may be further enhanced by catecholamine infusion, the current standard practice to maintain adequate perfusion pressure [6]. Potential therapies to mitigate mitochondrial oxidative stress range from enhancing antioxidant capacity to slowing down metabolic activity [7].

The so-called "third gaseous mediator", hydrogen sulfide (H_2_S), has various ubiquitous biological effects, among others a concentration-dependent modulation of mitochondrial activity, inasmuch as at low concentrations it may stimulate mitochondrial respiration, whereas high concentrations of H_2_S produce toxic mitochondrial inhibition [8, 9]. Furthermore, H_2_S has been demonstrated to attenuate oxidative and nitrosative stress during resuscitation from hemorrhagic shock [9–12]. Therefore, administration of H_2_S has been extensively studied in various murine models of hemorrhage and resuscitation, but so far results have been equivocal [9, 10, 13–16]. The potential pulmonary toxicity of inhaled H_2_S [17] and the generation of un-physiologically high-peak concentrations of H_2_S by sulfide salts [18] can be circumvented using sulfide donors that are already recognized drugs, e.g., ammonium tetrathiomolybdate or sodium thiosulfate (Na_2_S_2_O_3_) [7]. The thiosulfate anion (S_2_O_3_^2−^) is an endogenous oxidation product of H_2_S degradation, but, in turn, can also serve as a source of H_2_S, especially under hypoxic conditions, e.g., circulatory shock [19]. In fact, in murine acute lung injury induced by intra-tracheal endotoxin injection, intraperitoneal Na_2_S_2_O_3_ administration resulted in a several-fold increase of both plasma and lung tissue sulfide concentrations as assessed using fluorescent probes [20]. We confirmed this effect in a long-term porcine model of hemorrhage-and-resuscitation: continuous i.v. Na_2_S_2_O_3_ tripled blood sulfide concentrations as measured with combined gas chromatography/mass spectrometry [21]. In addition, Na_2_S_2_O_3_ was shown to replenish the antioxidant pool and, thereby, to exert mito-protective effects during liver I/R injury [22]. Finally, in mice with genetic deletion of the major vascular H_2_S-releasing enzyme, cystathionine-γ-lyase (CSE^−/−^), and, consequently, impaired endogenous H_2_S availability, Na_2_S_2_O_3_ improved pulmonary gas exchange, increased urine output and attenuated both circulatory failure and liver damage [23]. Therefore, in the present study, we tested the hypothesis whether Na_2_S_2_O_3_ might also exert organ-protective effects in non-genetically modified mice, i.e., well-preserved endogenous H_2_S availability. Given the above-mentioned potentially beneficial metabolic and antioxidant properties, we focused on energy metabolism by combining the quantification of metabolic pathways using stable, i.e., non-radioactive, isotope substrate labeling and the determination of mitochondrial respiration using "high-resolution respirometry" [23–25] together with the analysis of tissue expression of nitrotyrosine, a marker of combined oxidative and nitrosative stress [26, 27]. To strengthen the translational value of the study, all mice were studied using a "*post-treatment design*" in our well-established full-scale critical care model of resuscitated trauma-and-hemorrhage. 

## Methods

### Animals

The study had been approved by the University of Ulm Animal Care Committee and the Federal Authorities for Animal Research (Regierungspräsidium Tübingen, Baden-Württemberg, Germany, Reg.-Nr. 1387, approval January 31, 2018; subsequent approval for wild type mice dated March 11, 2021), and all experiments were performed in adherence with the National Institutes of Health Guidelines on the Use of Laboratory Animals and the European Union “Directive 2010/63 EU on the protection of animals used for scientific purposes”. The present study used a total of 30 male C57BL/6 J mice purchased from Charles River Laboratories (Wilmington, MA, USA) with an age of 20 (18–22) weeks and 32 (30–34) g body weight, respectively. Animals were kept under standardized conditions, with free access to water and food. Seven animals had to be excluded from the final data analysis due to hemo-pneumothorax and/or pericardial tamponade subsequent to blunt chest trauma, or uncontrollable bleeding during surgery. Thus, the subsequent data refer to 23 mice analyzed (vehicle group: *n* = 11; Na_2_S_2_O_3_ group: *n* = 12). The drop-out rate of 7/30 is similar to previous studies of our group using the same model [15, 23, 24] and is due to the extensive surgical instrumentation as well as the potential complications of the blast wave-induced blunt chest trauma.

### Anesthesia, surgical instrumentation, and experimental procedure

**Fig. 1:**
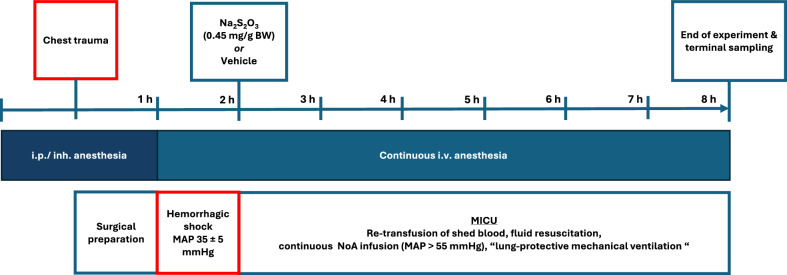
Experimental setup, timeline, surgical instrumentation, and experimental protocol (h hours; HS hemorrhagic shock; MAP mean arterial pressure; Na_2_S_2_O_3_, sodium thiosulfate; BW body weight; MIUC mouse intensive care unit; NoA noradrenaline)

Figure 1 shows the experimental setup, timeline, anesthesia, surgical preparation, and the experimental protocol. All experimental procedures, including anesthesia, surgical instrumentation as well as blast-wave induced blunt chest trauma, induction of hemorrhagic shock and resuscitation followed the same protocol as in previous reports [23–25], Initially, anesthesia was induced by s.c. administration of buprenorphine (1.5 µg/g_bodyweight_) as well as inhaled sevoflurane (2.5%). Immediately thereafter, mice underwent blunt chest trauma induced by a single blast wave centered on the thorax as established previously [28, 29]: In brief, compressed air rapidly ruptures a Mylar polyester film (Du Pont de Nemur, Bad Homburg, Germany), which releases a reproducible single blast wave toward the animal’s midsternal chest and thus induces a reproducible contusion of the lung. Then, all animals were connected to a closed loop body temperature control system (target temperature 37 °C) and placed on an instrumentation bench. Anesthesia was maintained by administration of 120 µg of S-ketamine i.p. (Ketanest-S, Pfizer, New York City, NY, USA), 0.25 µg/g_bodyweight_ of fentanyl (Fentanyl-hameln, Hameln Pharma Plus GmbH, Hameln, Germany) and 1.25 µg/g_bodyweight_ of midazolam (Midazolam-ratiopharm, Ratiopharm, Ulm, Germany) until central venous access was established. Surgical instrumentation included a tracheostomy and, thereafter, subsequent placement of catheters into the jugular vein, carotid and femoral artery as well as the urinary bladder. After tracheostomy, mice were mechanically ventilated following a lung-protective regimen (pressure-controlled ventilation, F_i_O_2_ 0.21; respiratory rate 150/min, tidal volume 6 µL/g_bodyweight,_ I:E 1:2). The positive end-expiratory pressure (PEEP) was set according to the ratio of arterial PO_2_/FiO_2_ (P/F), where PEEP was 3 cm H_2_O for P/F ratio > 300, 5 cm H_2_O for P/F < 300, and 8 cmH_2_O for P/F < 200. To prevent impairment of the thoraco-pulmonary compliance following anesthesia and supine positioning, recruitment maneuvers were performed hourly. After placement of the central venous catheter, anesthesia was provided intravenously by continuous infusion of 0.3 µg/g_bodyweight_/h of fentanyl and 30 µg/g_bodyweight_/h of esketamine. Upon completion of surgical procedures, hemorrhage was induced by removal of 30 µl blood/g_bodyweight_ via the femoral arterial line and adjusted to a mean arterial pressure of 35 ± 5 mmHg for 60 min by further blood removal or re-transfusion of blood (50 µL/bolus). Then, resuscitation was started by re-transfusion of shed blood combined with infusion of 20 µL/g_bodyweight_/h of hydroxyethyl starch (Tetraspan, Baun, Melsungen, Germany), and a continuous i.v. infusion of noradrenaline titrated as necessary to maintain mean arterial pressure (MAP) > 55 mmHg. Subsequent to the start of resuscitation, a bolus of 0.45 mg/g_bodyweight_ of Na_2_S_2_O_3_ or an equal amount of the vehicle solution NaCl 0.9% were administered according to the randomization protocol. The Na_2_S_2_O_3_ was chosen because it had improved pulmonary gas exchange, increased urine output and attenuated both circulatory failure and liver damage in a previous study, in which CSE^−/−^ mice underwent the identical experimental design for anesthesia, surgical instrumentation as well as trauma-and-hemorrhage and subsequent resuscitation [23]. Moreover, this Na_2_S_2_O_3_ dose had allowed replenishing the liver antioxidant pool in a murine model of hepatic I/R injury [22]. Experiments were completed by exsanguination and harvesting of blood and tissue samples after 6 h of intensive care or if MAP could no longer be maintained > 50 mmHg despite maximum noradrenaline infusion rates (i.e., 2 μg/kg_bodyweight_/minute) [23, 24].

### Parameters of hemodynamics, lung and kidney function, gas exchange, acid–base status

Hemodynamic parameters, core temperature and lung mechanics were obtained hourly using the LabChart System (ADInstruments, Colorado Springs, CO 80907, USA). Thoraco-pulmonary compliance was derived from respirator data (Flexivent small animal ventilator, Scirq, Montreal, QC, Canada). Arterial blood gas analyses including hemoglobin O_2_-saturation, acid–base status, lactate and glucose levels were performed at baseline as well as at the end of experiment. Plasma and urinary creatinine were assessed by gas chromatography/mass spectrometry (GC/MS) using ^2^H_3_-creatinine (CND isotopes, Pointe-Claire, QC H9R1H1, Canada) and used to calculate creatinine clearance according to the equation:

$$Creatinine \, clearance \, = \, Creatinine_{Urine} x_{{}} Volume_{Urine} / \, Creatinine_{Plasma} / \, time,$$

where Creatinine_Urine_, Creatinine_Plasma_, Volume_Urine_, and t are the urinary and plasma creatinine concentrations, the urine volume and urine sampling time, respectively.

### Stable isotope-based assessment of metabolic pathways

As reported in detail previously [25], endogenous glucose production rate and direct, aerobic glucose oxidation, as well as the production rates of urea, glycerol, and leucine as markers of the hepatic metabolic capacity, lipolysis, and protein degradation were derived from the plasma isotope enrichment during primed-continuous i.v. infusion of stable, non-radioactive isotope-labeled ^13^C_6_-glucose, ^15^N-urea, ^2^H_5_-glycerol (Campro Scientific, Berlin, Germany) and 5,5,5-^2^H_3_-leucine (CIL, Tewksbury, MA, USA). The direct, aerobic glucose oxidation rate was derived from the expiratory ^13^CO_2_ release after measuring the total expiratory CO_2_ release and the mixed expiratory ^13^CO_2_/^12^CO_2_ isotope enrichment. After thawing the plasma samples, they were spiked with internal standards (6,6-^2^H_2_-glucose, ^2^H_3_-creatinine, and N-methyl-urea for the quantification of glucose, creatinine, and urea), de-proteinized, and purified using cation-exchange solid-phase extraction (the separation of creatinine from interfering creatine and creatine phosphate). Glucose and glycerol were derivatized with N-methylbis(trifluoroacetamide) (MBTFA) to the corresponding trifluoroacetates. Urea and leucine were converted with N-(tert-butyldimethylsilyl)-N-methyltrifluoroacetamide (MTBSTFA) to tert-butyl-dimethylsilyl derivatives. Creatinine was analyzed as a trimethylsilyl derivative after a reaction with N,O-bis(trimethylsilyl)trifluoroacetamide (BSTFA). The GC/MS analysis were performed with an Agilent 6890/5973 GC/MS system using an Optima-5-MS capillary column (12 m × 0.2 mm i.d., 0.35 μm film thickness; Macherey–Nagel, Düren, Germany). The MS was operated under electron ionization, and metabolites were detected in the selected ion-monitoring mode. The plasma concentrations of glucose, urea, and creatinine as well as the urinary creatinine concentration were determined with a six-point calibration curve. The peak area ratios of the endogenous compound *vs.* the added internal standard were plotted against the amount ratios to generate calibration curves. The rates of appearances (endogenous glucose production, i.e., gluconeogenesis, glycerol, urea, and leucine production rates, respectively) were calculated from the isotope infusion rates and the measured ratio of labeled *vs.* unlabeled compounds in the plasma according to the formula

$$Ra \, = \, Infusion \, rate_{tracer} / \, TTR$$

where Ra is the rate of appearance, and TTR is the tracer (isotope-labeled compound)-to-tracee (endogenous compound) ratio. The glucose oxidation rates were calculated from the ^13^C_6_-glucose infusion rate, the total CO_2_ released, and the expiratory ^13^CO_2_ enrichments according to the formulae

$$Oxidation \, rate \, = \, V^{13} CO_{2} /^{13} C - infusion \, rate,$$

with

$$V^{13} CO_{2} = \, TTR -^{13} CO_{2} / \, \left( {TTR -^{13} CO_{2} + \, 1} \right) \, \cdot \, Content_{expiratory} - CO_{2} \cdot \, respiratory \, minute \, volume$$

where V^13^CO_2_ is the total expiratory ^13^CO_2_ release and TTR-^13^CO_2_ is the expiratory ^13^CO_2_/^12^CO_2_ ratio, respectively. For this purpose, the ^13^C-infusion rate was calculated as the product of the ^13^C_6_-glucose infusion times 6, i.e., the number of ^13^C-labeled C atoms in the glucose molecule.

### Tissue mitochondrial respiration

High-resolution respirometry using a Clark electrode-based systems (Oxygraph 2 k, OROBOROS Instruments Corp., Innsbruck, Austria) was used to quantify mitochondrial respiratory capacity in immediate *post mortem* liver and kidney tissue specimens as described previously [25]. In short, various states of mitochondrial function based on the use of different inhibitors or substrates were assessed in mechanically homogenized tissue samples. Specimens were homogenized in Mir05 (0.5 mM EGTA, 3 mM MgCl_2_ 6 H_2_O, 60 mM lactobionic acid, 10 mM KH_2_PO_4_, 20 mM HEPES, 110 mM sucrose, and 1 g/L bovine serum albumin) as a respiration medium and then added to the Oxygraph chamber (2 mg each of kidney and liver tissue). Subsequently to the addition of 10 mM pyruvate, 10 mM glutamate, 5 mM malate, 5 mM ADP, 10 µM cytochrome c,1 mM octanoyl-carnitine and 10 mM succinate, the maximum oxidative phosphorylation (OxPhos) was determined. Further addition of 2.5 µM oligomycin inhibited the ATP synthase and the uncoupling agent carbonyl cyanide-4(trifluoromethoxy)phenylhydrazone (FCCP, final concentration 1.5 µM) was added until the maximum respiratory activity of the electron transfer system in the uncoupled state (ETC) was reached.

### Tissue immunohistochemistry

Immunohistochemistry (IHC) was used to determine the expression of cystathionine-γ-lyase (CSE), albumin, and nitrotyrosine. IHC analysis was chosen, because Western blotting would yield limited information, since it would not allow evaluating the specific localization of the proteins of interest. Moreover, we previously showed that the protein quantification using IHC directly correlates with the quantification by Western blotting [30]. Moreover, Western blot analysis may be confounded by the presence of blood cells containing CSE [31]. Hence, Western blotting, a method requiring tissue homogenization, bears the risk yielding inaccurate results due to potential contamination. Clearly, protein quantification with IHC can be inaccurate when using fluorescent antibodies; therefore, we used a red chromogen and permanent mount, which prevents decaying of the signal. Immediately after euthanasia, the left kidney and the left liver lobe were sampled. The kidney was sagitally cut, and the capsule was removed. Tissue samples were then fixed in formalin, dehydrated and embedded in paraffin. IHC was performed on 3-μm sections after de-paraffinization in xylene and rehydration in graded mixtures of ethanol to de-ionized water. Heat-induced antigen retrieval was performed by microwaving in citrate buffer pH 6.0, followed by blocking with 10% normal goat serum, as described previously [32]. The following primary antibodies were used: cystathionine-γ-lyase (anti-CSE, rabbit polyclonal, Proteintech), albumin (anti-albumin, rabbit polyclonal, Proteintech), and nitrotyrosine (anti-nitrotyrosine, rabbit polyclonal, Merck Millipore). Primary antibodies were detected by a secondary antibody (goat-anti-rabbit IgG conjugated to alkaline phosphatase; Jackson ImmunoResearch, Cambridge, UK). Antibodies were visualized with a red chromogen (Dako REAL Detection System Chromogen Red, Dako, Glostrup, Denmark), and tissue was counterstained with Mayer’s hematoxylin (Sigma). Staining of representative 800,000 μm^2^ fields was evaluated with the Zeiss Axio Imager A1 microscope, a × 10 objective, and Zeiss Zen 3.0 (Zeiss, Oberkochen, Germany). Results are presented as % positive staining area.

### Statistics

Data were collected using Excel (version 16.97.2, Microsoft, Redmond, WA, USA). Statistical analysis was performed using Graphpad (version 9.5.1, Prism, Boston, MA, USA). Data are presented as either mean ± standard deviation or median (interquartile range) according to their respective distribution, which was tested using the Shapiro–Wilk test. Intergroup differences were evaluated using the Mann–Whitney *U* rank sum test (non-parametric distribution) or a Student’s *t*-test (parametric distribution). Differences within a treatment group were analyzed by a Wilcoxon rank sign test (non-parametric distribution) or a paired Student’s *t*-test. Survival differences between treatment arms were assessed by a log rank Mantel-Cox test. Limit for statistical significance was set to < 0.05. Sample sizes were based on the results of previous experiments using this murine full-scale ICU-model of resuscitation after combined blunt chest and hemorrhagic shock [23–25]. An independent statistical power analysis (by Prof. Rainer Muche, Institute of Epidemiology and Medical Biometry, University of Ulm: "*Tiergu185.doc*" dated October 17, 2017) had yielded a minimum number of 14–16 animals for the two experimental groups based on two-sided testing, *a* = 0.05, power of 80%, using as main criteria a doubling of the creatinine clearance (as marker of acute kidney injury) and a 50% reduction of the noradrenaline infusion rate (as marker of the severity of circulatory shock), respectively, when compared to the vehicle.

## Results

### Hemodynamics, lung and kidney function, gas exchange, acid–base status and survival

**Fig. 2:**
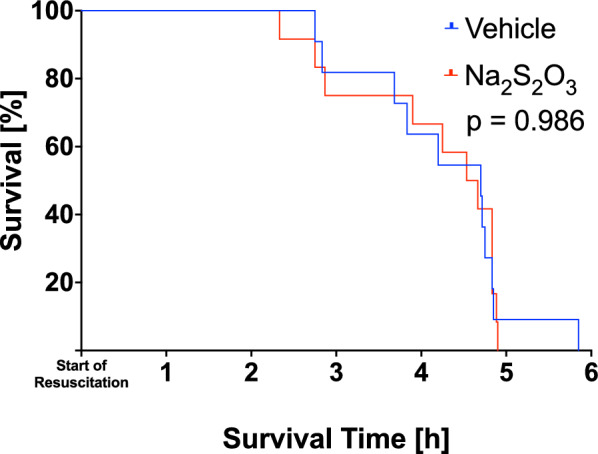
Kaplan–Meier survival curve in the vehicle (blue line) and Na_2_S_2_O_3_ (red line) groups with “Start of resuscitation” referring to “2 h” of the experimental time line (see Fig. 1). Survival times were strictly identical in the two groups (log rank Mantel–Cox test)

Table 1 summarizes the data referring to hemodynamics, gas exchange, lung mechanics, acid–base status, renal function and metabolic parameters at baseline, i.e., after induction of blunt chest trauma and the subsequent surgical instrumentation, and at the end of the experiment, i.e., after 6 h of resuscitation or, if necessary, pre-mature termination of the experiment. Due to the continuous noradrenaline infusion initiated upon the start of resuscitation, heart rate significantly increased in both groups (*p* < 0.001 each), while MAP was not significantly affected, however, without intergroup difference. Vasopressor requirements did not differ between the treatment groups either. As a result of the repetitive blood sampling and fluid resuscitation, total hemoglobin content significantly decreased over time (*p* < 0.001 in both groups), again without intergroup difference. While thoraco-pulmonary compliance and peak inspiratory pressures remained constant over time, minute ventilation had to be significantly increased in both groups (vehicle *p* = 0.019, Na_2_S_2_O_3_ *p* = 0.022) to maintain arterial PCO_2_ within the normal range. Arterial PO_2_ remained unaffected in the vehicle group, whereas it significantly decreased over time in the Na_2_S_2_O_3_-treated animals (*p* = 0.006), despite the adjustment of PEEP, which had to be significantly increased within this group until the end of the experiment (*p* = 0.015). Despite this different response patterns of arterial PO_2_, arterial hemoglobin O_2_-saturation decreased in both groups, albeit this effect did not reach statistical significance in the vehicle animals (vehicle *p* = 0.104, Na_2_S_2_O_3_ *p* < 0.003). The two experimental groups also presented with a significant time-dependent fall in arterial pH (vehicle *p* = 0.049, Na_2_S_2_O_3_ *p* < 0.001), which was more pronounced in the Na_2_S_2_O_3_–treated mice due to a significant aggravation of metabolic acidosis (*p* < 0.001). None of the other parameters showed any time-dependent or intergroup difference. Also, survival did not differ between the two experimental groups either (*p* = 0.986; Fig. 2).

**Table 1:** Hemodynamics, gas exchange, lung mechanics, acid–base status, renal function and metabolic parameters at baseline and at the end of the experiment

		Baseline	End of experiment
Heart rate [1/min]	Vehicle	328 ± 49.3	500 ± 117 #
	Na_2_S_2_O_3_	343 ± 29.8	530 ± 62 #
Mean arterial pressure [mmHg]	Vehicle	55 ± 13	64 ± 14
	Na_2_S_2_O_3_	50 ± 6	57 ± 8
Noradrenaline infusion rate [μg/kg/min]	Vehicle	n/a	0.33 [0.03–1.70]
	Na_2_S_2_O_3_	n/a	0.46 [0.30–0.70]
Hemoglobin	Vehicle	11.1 ± 1.0	6.6 ± 1.3 #
[g/dL]	Na_2_S_2_O_3_	11.6 ± 0.8	6.8 ± 0.7 #
Minute ventilation[mL/min]	Vehicle	27 ± 3	33 ± 3 #
	Na_2_S_2_O_3_	31 ± 4	37 ± 5 #
Positive end-expiratory pressure [cmH_2_O]	Vehicle	3 [3–3]	3 [3–5]
	Na_2_S_2_O_3_	3 [3–3]	5 [3–5] #
Peak inspiratory pressure [cmH_2_O]	Vehicle	8 [7, 8]	7 [6–10]
	Na_2_S_2_O_3_	8 [8–8]	9 [7–9]
Thoraco-pulmonary	Vehicle	106 [98–111]	99 [96–109]
compliance [µL/cm H_2_O]	Na_2_S_2_O_3_	94 [88–102]	110 [104–110]
Arterial PO_2_	Vehicle	85 ± 9	76 ± 12
[mmHg]	Na_2_S_2_O_3_	87 ± 13	65 ± 15 #
Arterial hemoglobin	Vehicle	91 [87–98]	79 [72–87]
O_2_ saturation [%]	Na_2_S_2_O_3_	90 [85–98]	72 [70–78] #
Arterial PCO_2_[mmHg]	Vehicle	35 [31–43]	36 [33–38]
	Na_2_S_2_O_3_	32 [28–39]	38 [35–40]
Arterial pH	Vehicle	7.35 ± 0.07	7.26 ± 0.08 #
	Na_2_S_2_O_3_	7.38 ± 0.06	7.19 ± 0.10 #
Arterial base excess[mmol/L]	Vehicle	− 4.8 ± 2.3	− 10.5 ± 4.6
	Na_2_S_2_O_3_	− 4.9 ± 2.5	− 11.9 ± 2.9 #
CO_2_ production	Vehicle	n.d	30 [24–31]
[μL/g/min]	Na_2_S_2_O_3_	n.d	31 [29–33]
Arterial glucose[mg/dL]	Vehicle	156 ± 22	132 ± 36
	Na_2_S_2_O_3_	149 ± 47	132 ± 34
Arterial lactate[mmol/L]	Vehicle	1.6 [1.2–2.3]	2.4 [1.5–5.3]
	Na_2_S_2_O_3_	1.5 [1.3–2.0]	2.1 [1.5–4.9]
Total urinary output	Vehicle	n.d	1,846 ± 1,1331
[µL]	Na_2_S_2_O_3_		495 ± 963
Plasma creatinine	Vehicle	n.d	2.0 ± 0.6
[μg/mL]	Na_2_S_2_O_3_		2.5 ± 1.0
Plasma urea	Vehicle	n.d	741 ± 251
[μg/mL]	Na_2_S_2_O_3_		719 ± 249
Creatinine clearance	Vehicle	n.d	314 ± 182
[μL/min]	Na_2_S_2_O_3_	n.d	257 ± 142

Data are presented as mean ± standard deviation or median [interquartile range], as appropriate. To test for differences between treatment groups, the Mann Whitney U rank sum test or a student’s t-test was used as appropriate. To test for differences between the two time points within a treatment group, a paired student’s t-test or a paired Wilcoxon rank sign test was used as appropriate. # depicts significant time-dependent differences within a group. There were no significant intergroup differences. *Of note*: "Baseline" values were obtained *after* blunt chest trauma and at the end of the surgical instrumentation, i.e., immediately after insertion of the arterial line with established deep, continuous i.v. anesthesia and under mechanical ventilation, but without continuous i.v. noradrenaline, which was only started upon the initiation of resuscitation

### Metabolic pathways

Table 2 shows the results of the stable isotope-based metabolic evaluations. Neither endogenous glucose production rate nor the fraction of direct, aerobic glucose oxidation significantly differed between the two experimental groups. The markers of the hepatic metabolic capacity, lipolysis, and protein degradation, i.e., the rates of appearance of urea, glycerol, and leucine did not significantly differ either.

**Table 2:** Results of the stable isotope-based evaluation of metabolic pathways

	Vehicle	Na_2_S_2_O_3_	p
Endogenous glucose production rate [µmol/g/h]	2.6 [2.6–4.3]	3.7 [3.4–4.2]	0.213
Glucose oxidation[% of infused isotope]	58 [44–64]	59 [47–61]	0.616
Urea production rate [µmol/g/h]	2.4 [1.7–2.7]	1.9 [1.8–2.2]	0.620
Glycerol production rate [µmol/g/h]	1.8 [1.4–3.3]	3.7 [2.6–4.2]	0.366
Leucine production rate [µmol/g/h]	0.5 [0.3–0.7]	0.5 [0.4–0.5]	0.748

Data are presented as median [interquartile range]. Neither endogenous glucose production rate nor the fraction of direct, aerobic glucose oxidation significantly differed between the two experimental groups. The markers of the hepatic metabolic capacity, lipolysis, and protein degradation, i.e., the rates of appearance of urea, glycerol, and leucine did not significantly differ either. Of note: After the priming bolus, a constant i.v. infusion time of at least 4 h is necessary to reach a steady state for plasma isotope enrichments. Therefore, data presented originate from n = 7 animals in the two groups only due to the premature termination of the experiment in the remainder animals (see survival, Fig. 2)

### Tissue mitochondrial respiration

Table 3 summarizes the results of the quantification of the tissue mitochondrial respiratory activity. Neither kidney nor liver maximum oxidative phosphorylation (OxPhos) or maximum respiratory activity of the electron transfer system in the uncoupled state (ETC) showed any significant intergroup difference.

**Table 3:** Results of the analysis of the liver and kidney mitochondrial activity O_2_ uptake (*J*O_2_) in pmol/s/mg_tissue_

		Vehicle	Na_2_S_2_O_3_	p
Kidney	OxPhos	363 [344–444]	323 [238–399]	0.432
	ETC	401 [347–484]	397 [215–439]	0.670
Liver	OxPhos	155 [107–202]	157 [92–217]	0.948
	ETC	228 [152–278]	160 [104–293]	0.854

Data are presented as median and interquartile range. Neither the oxidative phosphorylation in the coupled state (OxPhos) nor the maximal electron transfer capacity in the uncoupled state (ETC) showed any significant intergroup difference

### Tissue immunohistochemistry

**Fig. 3:**
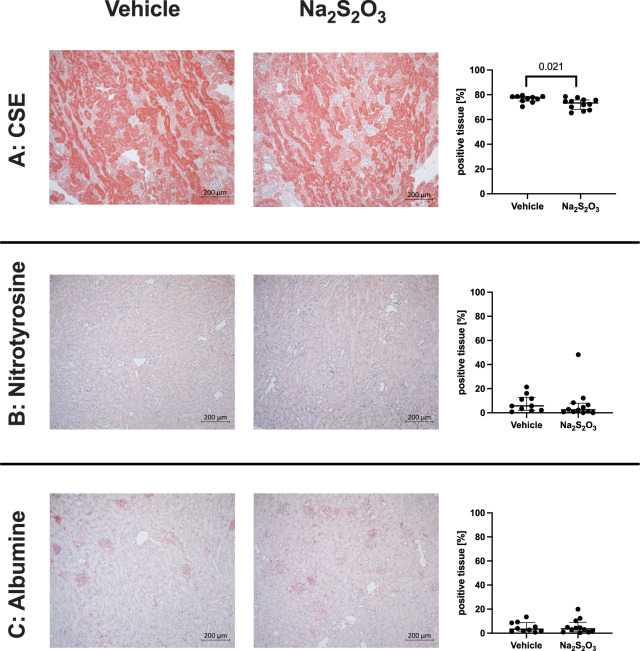
Examples (left panel) and quantitative evaluation for all individual animals of the immunohistochemistry analysis for kidney tissue cysatathionine-γ-lyase (CSE) (**A**), nitrotyrosine formation (**B**), and extravascular albumine (**C**) accumulation in the 2 experimental groups. Exemplary pictures are displayed with a magnification of 10x, quantitative data are presented as “% positive tissue staining”. Note that both nitrotyrosine formation and albumin accumulation were weak, indicating that there was only minor tissue oxidative/nitrosative stress and barrier disrupture

**Fig. 4:**
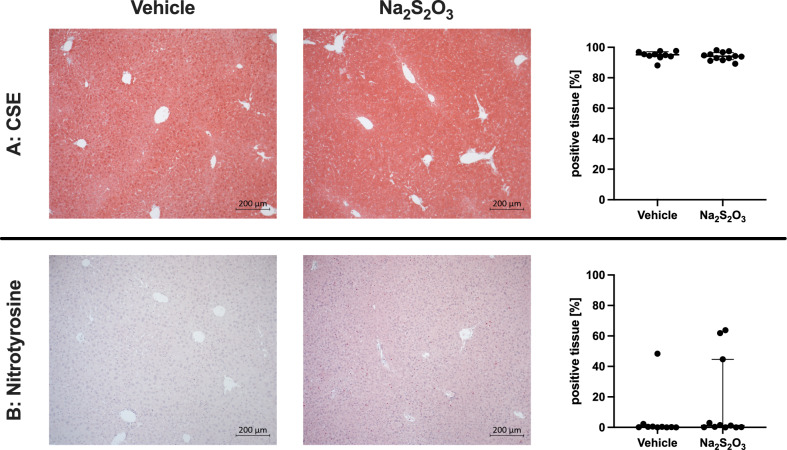
Examples (left panel) and quantitative evaluation for all individual animals of the immunohistochemistry analysis for liver tissue cysatathionine-γ-lyase (CSE) (**A**) and nitrotyrosine formation (**B**) in the 2 experimental groups. Exemplary pictures are displayed with a magnification of 10x, quantitative data are presented as “% positive tissue staining”. Note that there was only negligible nitrotyrosine formation, indicating that there was only minor tissue oxidative/nitrosative stress

Figures 3 and 4 show representative pictures (left panels) as well as the overall quantitative analysis (right panels) of the kidney (Fig. 3) and liver (Fig. 4) CSE expression (Figs. 3A and 4A), nitrotyrosine formation (Figs. 3B and 4B), and kidney extravascular albumin accumulation (Fig. 3C). Except for a slight, but significantly lower (*p* = 0.021) kidney CSE expression, Na_2_S_2_O_3_ treatment had no effect on any of these target proteins.

## Discussion

Here, we set out to determine the effect of Na_2_S_2_O_3_ as an adjunct to resuscitation in a murine model of trauma-and-hemorrhage. The main results were that (*i)* while Na_2_S_2_O_3_ affected neither hemodynamics nor kidney function, it even impaired lung function, without, however, any impact on survival; (*ii)* Na_2_S_2_O_3_ had no effect either on any of the metabolic pathways assessed nor on tissue mitochondrial respiratory activity, and (*iii)* failed to attenuate markers of tissue nitrosative stress and barrier dysfunction.

### Survival

Survival of mice was strictly identical in the two experimental groups. Our study was not powered to detect any differences in survival, but, nevertheless, our findings well agree with those previously observed in CSE^−/−^-mice with underlying co-morbidity (streptozotocin-induced diabetes type 1 [24] and pre-traumatic cigarette smoke exposure [25]) using the same model. Clearly, these findings are in contrast with previous murine studies, where Na_2_S_2_O_3_-treatment improved survival [33–35], but in none of these studies standard critical care measures had been integrated into the experimental design.

### Hemodynamics and organ function

Na_2_S_2_O_3_ treatment did not affect the noradrenaline infusion rates required to maintain the target hemodynamics. While this result contrasts with the findings of our previous study on CSE^−/−^ mice treated without co-morbidities [23], again it well agrees with our findings in CSE^−/−^ mice with pre-existing diabetes type 1 and pre-traumatic cigarette smoke exposure [24, 25]. Interestingly, in the vehicle group, the median NoA infusion rate was similar to that in our previous studies on CSE^−/−^ mice without co-morbidities and with diabetes type 1 (median: 0.33 *vs.* 0.27 [0.18–2.55] and 0.58 [0.12–0.89] μg/kg/min, respectively) [23, 24]. Moreover, the median MAP values at the end of the experiment were lower in the vehicle-group mice in these latter studies (48 and 44 mmHg in animals without co-morbidities and with diabetes type 1, respectively) than in the present study (64 mmHg). Finally, all the other factors potentially influencing systemic hemodynamics were also comparable in all three experimental series. However, the CSE^−/−^ strain studied in the previous experimental series is well-established to develop arterial hypertension as early as at the age of approx. 12 weeks [36]. We can only speculate on the reasons why both the wild type mice of the present study and the CSE^−/−^ animals of the previous experiments presented with comparable blood pressure values, but the continuous i.v. anesthesia and/or mechanical ventilation may have obscured any differences potentially present in awake animals.

Strikingly, Na_2_S_2_O_3_ treatment impaired both lung mechanics and pulmonary gas exchange, inasmuch as arterial PO_2_ decreased until the end of the experiment in this group, albeit the PEEP level had been increased as imposed by the experimental design. Taken together, these findings suggest increased intrapulmonary right-to-left shunting and/or ventilation/perfusion mismatch: clearly, we did not measure the total O_2_ consumption, but the strictly identical systemic hemodynamics suggest that the major extra-pulmonary factor influencing arterial PO_2_, i.e., the mixed venous PO_2_, did not assume major importance. We can only speculate about any further effect of Na_2_S_2_O_3_ treatment on dead space ventilation; the minute ventilation required to maintain the arterial PCO_2_ had to be increased over time, but this was necessary in the vehicle group as well.

Na_2_S_2_O_3_ administration was associated with a more pronounced fall over time in arterial base excess, but a similar albeit less pronounced effect was present in the vehicle groups as well. This observation is in clear contrast to all our previous studies on CSE^−/−^ mice using the same model, no matter the presence or absence of underlying co-morbidities [23–25]. However, it well agrees with our previous studies in swine, where a continuous i.v. infusion of Na_2_S_2_O_3_ over 24 h had been associated with a moderate metabolic acidosis [21, 37]. Since lactatemia was virtually identical in the two experimental groups, this Na_2_S_2_O_3_-related acidosis was most likely due to an increased anion gap, such as reported in clinical case reports [38, 39]. It is noteworthy in this context that metabolic acidosis was present already at baseline and progressively aggravated in both experimental groups. In fact, in mechanically ventilated mice even without any further challenge, several other authors have previously observed metabolic acidosis of unknown origin [40–43], eventually even prompting compensation with continuous NaHCO_3_ infusion [44, 45]. This metabolic acidosis most likely also caused a right-shift of the Hb-O_2_-dissocation curve: the measured arterial hemoglobin O_2_-saturation values were lower than those which would have been predicted from the simultaneously recorded PaO_2_-levels.

### Metabolic pathways and tissue mitochondrial respiration

In good agreement with our previous experiments using the same model in CSE^−/−^ mice, Na_2_S_2_O_3_ did not affect tissue mitochondrial respiratory capacity. This is in clear contrast to data reported by other authors, who found mito-protective properties of Na_2_S_2_O_3_ after liver I/R injury [22]. It should be noted, however, that, similar to other experiments demonstrating therapeutic effects of Na_2_S_2_O_3_ in murine models [33–35], no standard critical care measure had been integrated into the experimental design. In addition to the lacking effect on tissue mitochondrial respiration, we could neither confirm the increased direct, aerobic glucose oxidation rate observed in animals of this strain with pre-traumatic cigarette smoke exposure [25] nor the reduction of endogenous glucose production rate, i.e., the rate of gluconeogenesis, in CSE^−/−^ mice without underlying co-morbidity [23]. In other words, despite a thorough investigation of various metabolic pathways using sophisticated, stable isotope-based analysis of whole-body metabolism rather than only measuring blood substrate concentrations, we could not find any metabolic effect of the Na_2_S_2_O_3_ treatment in this model. Consequently, it is most likely that a beneficial impact on metabolism of Na_2_S_2_O_3_ as most recently suggested for I/R injury [7] may only be detectable in the presence of impaired endogenous H_2_S availability, e.g., in CSE^−/−^ as in our previous experiments.

### Tissue markers of oxidative/nitrosative stress and barrier function

Except for the slight, but significantly lower kidney CSE expression, Na_2_S_2_O_3_ treatment had no effect on nitrotyrosine formation or extravascular albumin accumulation, i.e., markers of both oxidative and nitrosative stress [26, 27] and barrier dysfunction, respectively [46]. This finding contrasts with data on H_2_S supplementation using the "slow H_2_S-releasing" H_2_S donor GYY4137, which attenuated kidney nitrotyrosine formation in murine diabetic nephropathy [47, 48]. Moreover, our observation also contrasts with the marked attenuation of oxidative damage achieved by both the H_2_S-releasing salt Na_2_S and Na_2_S_2_O_3_ in murine liver I/R injury [22]. It is tempting to speculate that the full scale intensive care treatment in our present study, in particular the continuous i.v. noradrenaline, which is well-established to aggravate oxidative stress [49, 50], may have prevented any beneficial effect of Na_2_S_2_O_3_ on antioxidant capacity. Nevertheless, our findings do agree with our previous study in swine, where we did not find any effect of Na_2_S_2_O_3_ treatment on plasma 8-isoprostane or nitrite + nitrate concentrations nor superoxide dismutase activity, i.e., markers of oxidative stress, nitric oxide release and antioxidant capacity, respectively [21, 37]. Na_2_S_2_O_3_ did not affect kidney extravascular albumin accumulation either, a well-established marker of tissue barrier dysfunction. Again, this finding well agrees with previous studies: despite a significant improvement in lung mechanics and gas exchange, Na_2_S_2_O_3_ administration in swine with impaired endogenous H_2_S availability due to ubiquitous atherosclerosis affected neither lung tissue nitrotyrosine formation nor albumin accumulation [21].

Interestingly, Na_2_S_2_O_3_ administration was associated with a slight, but significantly lower CSE expression in the kidney, whereas no difference was found in the liver. Clearly, even in the Na_2_S_2_O_3_ treated mice, kidney CSE expression was still positive in 73% (*vs.* 77% in the vehicle group) of the tissue area analyzed, thus the biological significance of this finding is questionable. Nevertheless, our finding is in contrast with a recent report on the effects of Na_2_S_2_O_3_ treatment on CSE expression in juvenile, hypertensive rats with chronic kidney disease: 6–9 weeks of sustained Na_2_S_2_O_3_ administration was associated with even increased *kidney* CSE expression [51]. Moreover, in rats with myocardial I/R injury, Na_2_S_2_O_3_ administered upon the start of reperfusion also increased *cardiac* CSE expression [52]. However, similar to our study, the increased endogenous thiosulfate concentrations in endotoxin-challenged mice did not affect *liver* CSE expression. Dosing and our timing of the Na_2_S_2_O_3_ treatment as well as again, the full-scale intensive care treatment may be responsible for these discrepancies.

### Limitations of the study

It could be argued that we should have designed a longer period of full-scale ICU comprising mechanical ventilation, fluid resuscitation and continuous i.v. noradrenaline, rather than the maximum of six hours in the present study. It must be underscored, however, that the vast majority of studies using mechanical ventilation in mice, even when lung-protective ventilator settings were used and recruitment maneuvers were performed as in this study, are also limited to six hours of mechanical ventilation [53]. Clearly, there are reports available in the literature extending mechanical ventilation in mice up to eight or even twelve hours in combination with hemodynamic monitoring [44, 45, 54–58]. However, in none of these studies, mice additionally underwent a period of circulatory shock, and, subsequently, noradrenaline treatment to restore target hemodynamics.

It is unlikely that combining chest trauma with hemorrhagic shock may have mitigated any potential therapeutic effect of Na_2_S_2_O_3_ when compared to hemorrhagic shock alone as a result of more severely impaired gas exchange: in our various previous murine studies on the effects of blunt chest trauma or hemorrhagic shock alone and the combination of these two challenges, respectively, we did not find major differences in static thoraco-pulmonary compliance or pulmonary gas exchange unless a pre-existing pathology, e.g., cigarette smoke-exposure COPD, was present and or genetically modified mouse strains were investigated [15, 16, 23, 28, 29, 59, 60].

Clearly, this study lacks a Na_2_S_2_O_3_-treated group without hemorrhage and shock to comparatively explore the effects of Na_2_S_2_O_3_ in healthy animals. However, to adhere to the 3R principle, the federal authorities only allowed for the groups presented in this analysis.

Finally, since only one animal of the vehicle group and none of the Na_2_S_2_O_3_-treated animals survived until the programmed end at six hours of resuscitation, the combined challenge in this model per se may have been too severe to allow for detecting a therapeutic benefit of the Na_2_S_2_O_3_ treatment. The survival curves, however, are similar, to our previous experiments testing Na_2_S_2_O_3_ in CSE^−/−^ mice with pre-existing co-morbidities [24, 25]. A higher survival rate had only been observed, when Na_2_S_2_O_3_ was used CSE^−/−^ mice without any pre-existing co-morbidities [23].

## Conclusion

Previous studies supporting a beneficial role of Na_2_S_2_O_3_ suffered from either a lack of common critical care resuscitation or were carried out in CSE-deficient animals, in which an underlying impairment of endogenous H_2_S availability might have allowed for therapeutic effects. In the present analysis using a murine trauma-and-hemorrhage model including full scale critical care measures, administration of Na_2_S_2_O_3_ failed to improve survival and hemodynamic stability. Moreover, Na_2_S_2_O_3_ was even associated with impaired respiratory function and might have contributed to deteriorating metabolic acidosis. Extensive evaluation of metabolic pathways, mitochondrial function as well as markers of oxidative stress and kidney barrier dysfunction did not suggest any beneficial effect of Na_2_S_2_O_3_ either. Although a beneficial role of Na_2_S_2_O_3_ has been suspected for trauma resuscitation, our results in non-genetically modified mice agree—in general—with findings about Na_2_S_2_O_3_ administration in trauma-and-hemorrhage models using CSE deficient mice with and without pre-existing co-morbidities. In current critical care, patients with pre-existing comorbidities are common, while sufficient CSE and, hence, adequate peripheral H_2_S levels might be assumed. Following this in combination with the findings presented in the current study, the role of Na_2_S_2_O_3_ as an adjunct to trauma resuscitation in a critical care setting appears questionable.

## Data Availability

The datasets used and/or analyzed during the current study are available from the corresponding author on reasonable request.
